# Beyond antibiotics: CRISPR/Cas9 triumph over biofilm-associated antibiotic resistance infections

**DOI:** 10.3389/fcimb.2024.1408569

**Published:** 2024-07-05

**Authors:** Azna Zuberi, Nayeem Ahmad, Hafiz Ahmad, Mohd Saeed, Irfan Ahmad

**Affiliations:** ^1^ Department of Molecular, Cellular & Developmental Biology, University of Colorado Boulder, Boulder, CO, United States; ^2^ Department of Obs & Gynae, Northwestern University, Chicago, IL, United States; ^3^ Department of Biophysics, All India Institute of Medical Science, New Delhi, India; ^4^ Department of Microbiology, Immunology, and Infectious Diseases, College of Medicine and Medical Sciences, Arabian Gulf University, Manama, Bahrain; ^5^ Department of Medical Microbiology & Immunology, Ras Al Khaimah (RAK) College of Medical Sciences, Ras Al Khaimah (RAK) Medical and Health Sciences University, Ras Al Khaimah, United Arab Emirates; ^6^ Department of Biology, College of Science University of Hail, Hail, Saudi Arabia; ^7^ Department of Clinical Laboratory Sciences, College of Applied Medical Sciences, King Khalid University, Abha, Saudi Arabia

**Keywords:** antibiotic resistance, biofilm, CRSISR/Cas9, bacteria, gene editing, infections

## Abstract

A complex structure known as a biofilm is formed when a variety of bacterial colonies or a single type of cell in a group sticks to a surface. The extracellular polymeric compounds that encase these cells, often consisting of proteins, eDNA, and polysaccharides, exhibit strong antibiotic resistance. Concerns about biofilm in the pharmaceutical industry, public health, and medical fields have sparked a lot of interest, as antibiotic resistance is a unique capacity exhibited by these biofilm-producing bacteria, which increases morbidity and death. Biofilm formation is a complicated process that is controlled by several variables. Insights into the processes to target for the therapy have been gained from multiple attempts to dissect the biofilm formation process. Targeting pathogens within a biofilm is profitable because the bacterial pathogens become considerably more resistant to drugs in the biofilm state. Although biofilm-mediated infections can be lessened using the currently available medications, there has been a lot of focus on the development of new approaches, such as bioinformatics tools, for both treating and preventing the production of biofilms. Technologies such as transcriptomics, metabolomics, nanotherapeutics and proteomics are also used to develop novel anti-biofilm agents. These techniques help to identify small compounds that can be used to inhibit important biofilm regulators. The field of appropriate control strategies to avoid biofilm formation is expanding quickly because of this spurred study. As a result, the current article addresses our current knowledge of how biofilms form, the mechanisms by which bacteria in biofilms resist antibiotics, and cutting-edge treatment approaches for infections caused by biofilms. Furthermore, we have showcased current ongoing research utilizing the CRISPR/Cas9 gene editing system to combat bacterial biofilm infections, particularly those brought on by lethal drug-resistant pathogens, concluded the article with a novel hypothesis and aspirations, and acknowledged certain limitations.

## 1 Introduction

It has long been known that microbiological infections exist, and since then, scientists have worked tirelessly to eradicate both established and new infections that cause infectious diseases and create antimicrobial drugs to cure and get rid of the contagious illness. Antimicrobials are a diverse group of substances that can fight a broad range of pathogenic microbes, including bacteria, protozoa, fungi, viruses and parasites (Dutt et al., 2022; Ahmad et al., 2023). From the early 20^th^ century, these substances have been employed to treat infected individuals and they have greatly assisted in reducing the most infectious rates of morbidity and mortality (Dutt et al., 2022). In 1928, penicillin was discovered by Alexander Fleming, and it entered clinical usage in the 1940s at the perfect moment for the second world war (Gaynes, 2017). In just four years of usage, the first strains of penicillin-resistant bacteria, a new species of bacteria appeared, which led to the development of antibiotic resistance. As a result, AMR has quickened and expanded to include more harmful species because of the continuous exposure and indiscriminate use of antibiotics in clinical and farming environments. Numerous modern antibiotics have lost their efficacy because of the development of AMR and scientists are working continuously to comprehend how AMR works to create new antimicrobials (Dutt et al., 2022).

Looking towards bacteria, it feels that the capacity to form biofilm is shared by mostly all the bacteria and it is considered a universal attribute. In biofilms, the extracellular matrix formed by the cells themselves holds the groups of bacteria or multicellular communities together. Different bacteria use different ways to create biofilms, these mechanisms soften depending on the environment in which they are found as well as strain-specific characteristics (Percival et al., 2011). He was Antonie van Leeuwenhoek who first saw “animalcules “on his teeth in the 17^th^ century. The bottle effect was first noted in marine microorganisms in 1940. This demonstrates that germs proliferate more frequently on surfaces (Percival et al., 2011; Dutt et al., 2022).

Then, in 1943, Zobell created biofilms and discovered that the number of bacteria on surfaces was higher than that of the surrounding seawater (Zobell, 1943). Today, in scientific language, we define biofilms as the microbial communities that are attached to a substrate and covered in extracellular polymeric substance (EPS), which is secreted by these bacteria (Costerton et al., 1987; Donlan, 2001; Zuberi et al., 2017b). Microbial biofilms can be found on many surfaces in aquatic environments, damp structures, plant roots, human tooth or dental implants, catheters, medical equipment, sutures, etc. They can even be found in human and animal tissues in pathogenic forms that can release toxins into the surrounding extracellular matrix (Donlan, 2001; Percival et al., 2011). They can evade the human response (Cangui-Panchi et al., 2022, Cangui-Panchi et al., 2023).

In addition, biofilms can be found in symbiotic form in aquatic bodies, wastewater filters and the alimentary canals of humans and animals (Costerton et al., 1981; Dutt et al., 2022). Because of their resistance to antibiotics, biofilms, which are the most common in natural settings, can infect both humans and animals (Mah, 2012; Sinclair, 2019). Thus, it is crucial to comprehend the mechanism of biofilm-led resistance to antibiotics. This review will cover antimicrobial resistance (AMR), specifically the mechanism underlying biofilm-led AMR, possible pharmacological or drug candidates and present modalities that are used to target bacteria within the biofilm. We will also focus on current ongoing research like the CRISPR/Cas9 gene editing system to fight bacterial biofilm infections.

## 2 Revisiting bacteria biofilm ultrastructural and its antibiotic survival strategies

Bacterial biofilms are groups of sessile microbes that are entrenched and connected to substratum, within the self-generated non-crystalline pool of the extracellular matrix (Zuberi et al., 2017a, Zuberi et al., 2017b). These bacterial communities are distinct from planktonic ones concerning several methods like transcription, gene expression and growth rate since they live in various stressed environments with increased cell density, osmolarity, nutrient shortage, etc (Sharma et al., 2019). Bacterial biofilm is a dynamic three-dimensional structure that is processed by a heterogenous group of bacterial communities and the bacteria living in these dynamic strictures are shielded from many environmental stressors, including desiccation, immune attack, protozoan ingestion, antimicrobial target, etc., hence making them more resistant and superior over the planktonic form of bacteria (Wilkins et al., 2014; Sharma et al., 2019). The process of developing this three-dimensional structure is a multi-step procedure. It begins when bacteria first attach themselves to the surface and create an unbreakable bond that is followed by other bacterial colonization (Sharma et al., 2019). The bacteria that colonize first are called primary colonizers in biofilm and the secondary colonizers are the ones that attach to the primary colonizers (MaChado and Cerca, 2015). This stage led to changes in the expression of genes or proteins by bacteria. The second stage of the exponential growth phase is characterized by the secretion of exopolysaccharides and the creation of water channels by these bacteria, enabling the nutrient supply to mature biofilms. Finally, the cell surface detachment begins the relaunched or recycled biofilm development on the fresh surfaces (Sharma et al., 2019). This detachment step is usually triggered by stress factors such as a limited nutritional environment and antibiotics. Usually, the cells in the inner or deep layers of biofilm become latent or dormant cells, while the ones on the top layers are metabolically active.

Biofilms can host multiple bacterial species and can lead to a complex system that can accommodate bacterial cell densities between 10^8^–10^11^ cells g^-1^ wet weight (Morgan-Sagastume et al., 2008). Water makes up to 97% of its matrix major constituents and the other contents include proteins, soluble or gel-forming polysaccharides, extra-cellar DNA (eDNA) and non-soluble elements like cellulose, amyloids, pili, fimbria and flagella that deliver structural and functional properties to these biofilms (Flemming and Wingender, 2010; Flemming et al., 2016).

Because of their increased resistance to antibiotics and disinfectants, bacterial biofilms are a major contributing factor to chronic infections. They can also interfere with phagocytosis and other immune system functions. As a result, microorganisms within biofilms become less vulnerable to various antibiotic medicines, hence posing an imminent challenge in the field of therapeutics (Høiby et al., 2010; Amato et al., 2014; Flemming et al., 2016; Dutt et al., 2022).

## 3 The elucidation of mechanisms governing bacterial biofilm resistance and its dynamics

The term antimicrobials is used for substances that kill microorganisms, inhibit their growth, and prevent or treat diseases or infections in animals, humans, and plants. They included a wide variety of antivirals, antifungals, and antiparasitics. The capacity of microorganisms to withstand an antimicrobial at a higher dose for an extended duration is known as antimicrobial resistance and is usually measured in terms of its minimum inhibitory concentration (Brauner et al., 2016; Ahmad et al., 2017, Ahmad et al., 2018). In biofilms, antibiotic tolerance or resistance may occur simultaneously. Through the introduction of foreign genetic material that codes for resistance genes by horizontal gene transfer (HGT) between the bacterial cells of the biofilm or through genetic mutation, microorganisms within the biofilm develop antibiotic resistance. As a subset of antimicrobial resistance (AMR), antibiotic resistance (ABR) occurs when bacteria develop resistance to antibiotics despite the drug’s effectiveness against them (Dutt et al., 2022). Usually, antibiotic resistance can be acquired extrinsically, adaptively or intrinsically. The classic example of intrinsically acquired resistance is the susceptibility of gram-positive bacteria against antibiotics like daptomycin or vancomycin over gram-negative bacteria due to the difference in their cell wall composition. Conversely, acquired resistance results from either mutation or horizontal gene transfer (HGT). In addition, during adaptation, the bacteria may quickly modify their pattern of gene expression and translation in response to other environmental conditions or stimuli including stress (Blair et al., 2015; Dutt et al., 2022).

**Figure 1:**
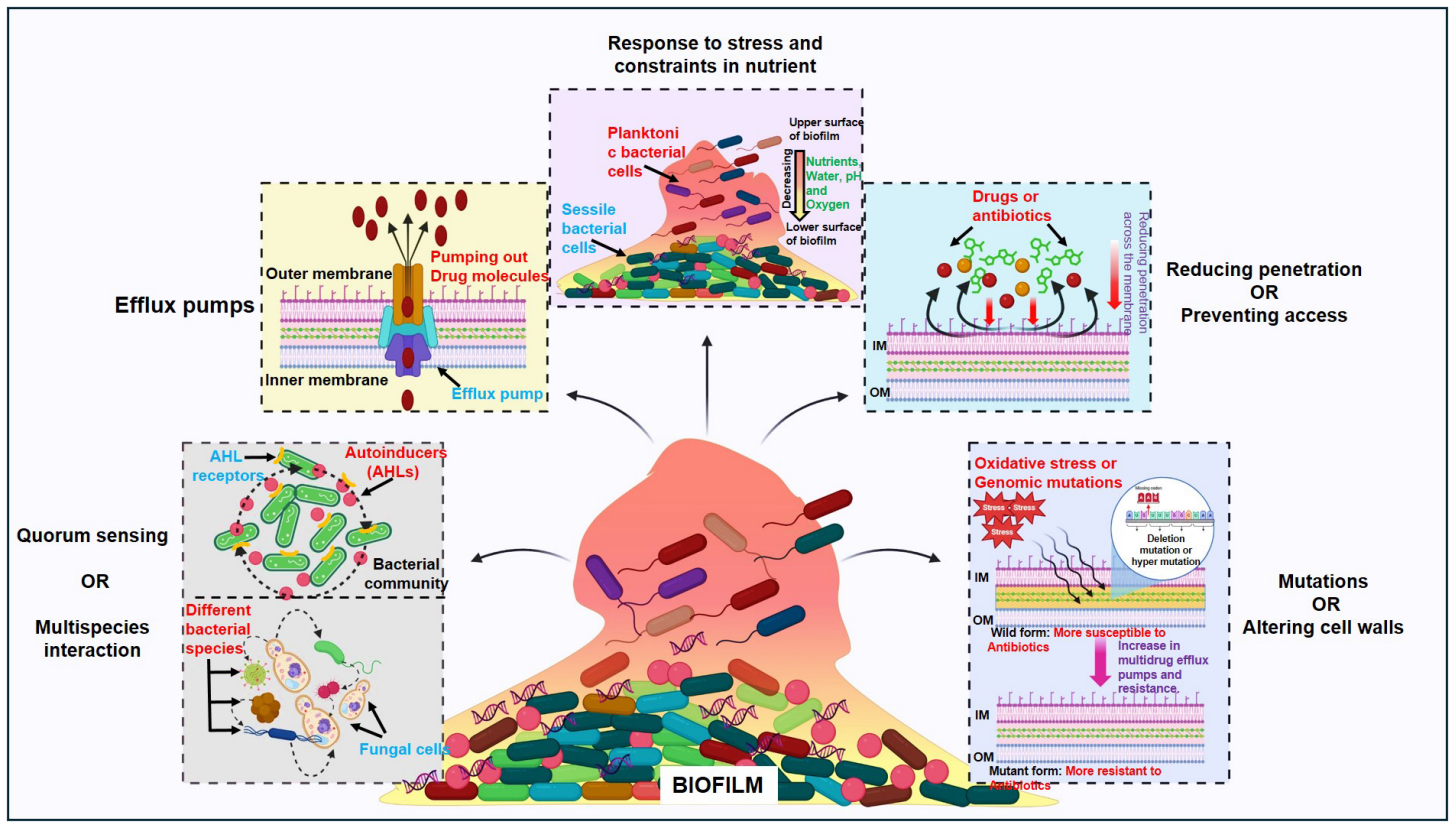
Regulatory pathways controlling biofilm bacterial resistance: Quorum sensing or multispecies interaction, efflux pumps, response to stress and constraints in nutrients, reducing penetration or preventing access and mutations or altering cell walls.

On the other hand, tolerance refers to a microorganism’s capability to endure antibiotics at concentrations greater than their inhibitory effect for a specific period (Arzanlou et al., 2017; Hall and Mah, 2017). For a brief duration, tolerance is a form of adaptation that represents shifts in cellular activity from active to latent state. Like antibiotic entrapment to the extracellular polymeric substance (EPS) in the absence of target attachment induces tolerance and causes bacteria cell dormancy. Persistence is an exceptional form of tolerance, where persisters refer to the tolerant form of cells within that bacterial population that can survive the antibiotics but can be killed at long exposure (Wilmaerts et al., 2019). Biofilm-mediated resistance is a multifaceted type of resistance that necessitates tolerance in addition to the antibiotic resistance mechanism. Furthermore, the state and developmental stage of the biofilm, its growth conditions, and the microbial species present within it also contribute to this process (Hall and Mah, 2017; Dutt et al., 2022). Some of the mechanisms of antimicrobial resistance (AMR) that are related to biofilm-mediated resistance are limiting the permeability or blocking access to antimicrobials; altering the targets of antibiotics through mutations; and breaking down the antimicrobials through enzymatic hydrolysis or chemical alteration. In this review, we tried to discuss each of them under the following headings and they have been vividly illustrated in **Figure 1**.

### 3.1 Quorum sensing and multispecies interaction

Bacterial; cells communicate with each other through a mechanism called quorum sensing that includes the synthesis, section, and reaction in response to the extracellular signalling molecules that are called autoinducers (AIs). As bacterial population density rises, these AIs build up the environment, and the bacterial cell uses this to carry out the actions that are advantageous when carried out by bacterial colonies operating simultaneously (Arevalo-Ferro et al., 2003; Di Cagno et al., 2011; Rutherford and Bassler, 2012; Zuberi et al., 2017b). Bioluminescence, competence, biofilm synthesis and virulence factor synthesis are some of the major processes regulated by QS (Rutherford and Bassler, 2012). To start the process of gene transcription for surface proteins, virulence proteins or proteins related to biofilm formation, these auto-inducers are secreted by bacteria and identified by other bacteria by their cell surface receptors. The main types of autoinducers include the acyl-homoserine lactones (AHL) that are found in Gram-negative bacteria, secondarily modified oligopeptides that are associated with Gram-positive bacteria, and a class of 4,5-dihydroxy-2,3-pentonedione-derived signal molecules termed autoinducer-2 (AI-2) that are found in both Gram-negative as well as the Gram-positive bacteria (Li and Nair, 2012). It has been demonstrated by different studies that the bacteria-bearing genes related to quorum sensing like *luxS, lasR*, and *rhlR* are more resistant to the antibacterial treatment (Dutt et al., 2022). The classic example comes from *P. aeruginosa* biofilms lacking genes *rhlR* and *lasR*, which were found more tobramycin-sensitive than their biofilms of the wild type (Bjarnsholt et al., 2005). Similarly, it was found that *S. aureus* lacking QS-specific *agrD* was less resistant than its matched wild-type (Yarwood et al., 2004). Moreover, *E. faecalis fsrA* and *gelE* mutants for quorum sensing and its controlled protease were less able to produce biofilms when antibiotics like gentamycin or daptomycin were present (Dale et al., 2015).

Multispecies interaction is another factor that drives antibiotic tolerance, for instance, it was found that the polymicrobial biofilms of *Finogoldia magna*, *S. aureus* and *E. faecalis* were twice as resistant as *P. aeruginosa* mono-species biofilm (Dalton et al., 2011). Similarly, *M. catarrhalis* released beta-lactamase in a dual-species model that shielded S. pneumonia against amoxicillin (Budhani and Struthers, 1998; Perez et al., 2014). Additionally, research has been done on the relationship that develops among fungi and bacteria in a multispecies biofilm. In *C. albicans* and *S. aureus* biofilms, the fungal matrix ingredient beta-1,3 glucan, which is thought to function as a barrier against vancomycin, increased the resistance of *Staphylococcus* to the antibiotic (Adam et al., 2002; Harriott & Noverr, 2009). Additionally, it was discovered that C. albicans can produce more alcohol, which in turn increases *P. aeruginosa* biofilm development (Chen et al., 2014).

### 3.2 Efflux pumps

Nearly all bacterial species contain efflux pumps. Bacterial efflux pumps are grouped into five families depending on attributes such as composition, substrates, energy sources and several transmembrane-spanning regions. They include the resistance-nodulation-division (RND) family, the ATP (adenosine triphosphate)-binding cassette (ABC) superfamily, the major facilitator superfamily (MFS), the multidrug and toxic compound extrusion (MATE) family and the small multidrug resistance (SMR) family (Sun et al., 2014). This process gives bacterial cells resistance because it transfers a drug in and out of the cell despite adhering to an intracellular target (Poole, 2007). According to certain theories, planktonic resistance in *P. aeruginosa* to low concentrations of ofloxacin is caused by several multidrug efflux pump systems including MaxAB-OprM (Brooun et al., 2000). It is thought that PA1875–1877, a significant multidrug efflux pump, plays a role in the biofilm resistance of *P. aeruginosa* (Zhang and Mah, 2008). The deletion of PA1875, PA1876 and PA1876 increased the sensitivity of biofilm to certain antibiotic drugs like gentamicin, ciprofloxacin and tobramycin by two to four times; however planktonic cells susceptibility was not significantly impacted (Zhang and Mah, 2008). Likewise, it was also claimed that the MexCD-OprJ or MexAB-OprM efflux pumps were also the cause of *P. aeruginosa* biofilms resistance to azithromycin (Gillis et al., 2005; Pamp et al., 2008).

### 3.3 Response to stress and constraints in nutrition

The Gradients of water, nutrients, pH, waste product dispersion and signalling molecules are usually dependent and determined by the three-dimensional dynamic structure and architecture of biofilms (de Beer et al., 1994a; Anderl et al., 2000; Borriello et al., 2004; Stewart and Franklin, 2008; Williamson et al., 2012; Stewart et al., 2016). It is seen that the cells close to the surface of a biofilm microcolonies use up most of the available nutrients, creating an impoverished region deeper down, that develops a variety of psychological states including anaerobic, microaerobic, aerobic and fermentative conditions (Stewart and Franklin, 2008; Flemming et al., 2016; Dutt et al., 2022). This process also affects the growth of those underlying cells and leads to dormant cells due to the scarcity of oxygen and nutrients in the lower layers of biofilm. In the other study, they discovered a unique characteristic when they cultured multidrug resistance *E. faecalis* strains straight from the biofilm stage after they had been stored at 70°C for 16 to 18 months. According to them, the re-cultured bacterial cells were found only to grow after 60–72 hours of incubation at 37°C and they turned out to be extra resistant to the antibiotics (Borriello et al., 2006). Tolerance to antibiotics that target the protein synthesis process or DNA gyrase like ciprofloxacin or tobramycin is thought to arise from the lower metabolic activity and possible dormancy in cells in the oxygen-deprived area of biofilms (Borriello et al., 2006; Dutt et al., 2022).

It is well known that colistin, which works on the cell membrane, can damage slowly regrowing cells (Haagensen et al., 2007). On the other hand, the observation of colistin-tolerant cells in oxygen-rich locations causes doubts about the relationship between antibiotic tolerance and the slow growth rate of cells (Pamp et al., 2008; Chiang et al., 2012). Yogesh and Anjali investigated this fact in 2021, discovering that colistin did not affect biofilm-forming *E. faecalis* (Dutt et al., 2022). In regions with high levels of oxygen, observable cellular activity and protein production have also been seen (Walters et al., 2003; Werner et al., 2004). *P. aeruginosa* can maintain anaerobic conditions through denitrification and fermentation. Supplementing nitrate or L-arginine might boost metabolic activity in nutrient-deprived regions, making them more susceptible to tobramycin as well as ciprofloxacin (Borriello et al., 2006).

### 3.4 Reducing penetration or preventing access

The structure and makeup of the extracellular matrix (ECM) can significantly impact antibiotic penetration, its entry into cells and ultimately, antibiotic effectiveness through gradients of dispersion (de Beer et al., 1994b). Antibiotic diffusion is also dependent on its interaction with the ECM constituents. Extracellular DNA, for instance, strengths *Pseudomonas* biofilm resistance to aminoglycosides but not to fluoroquinolones or beta-lactam antibiotics (Mulcahy et al., 2008; Doroshenko et al., 2014; Wilton et al., 2016). Similarly, eDNA strengthens the *Staphylococcus* epidermis biofilm’s ability to resist glycopeptides. It has been observed that negatively charged aminoglycosides (tobramycin) and glycopeptides (vancomycin) bind to negatively charged eDNA (Doroshenko et al., 2014). Additionally, it has been shown that the interaction between eDNA and vancomycin is 100 times stronger than that between peptides D-Ala-D-Ala and vancomycin in peptidoglycan precursors, that could lead to build-up eDNA in the ECM (Doroshenko et al., 2014). A multi-species biofilm can also contain antibiotic-modifying enzymes that can be released and found in the extracellular matrix (ECM), which other susceptible bacterial species can employ. As an example, Moraxella catarrhalis releases beta-lactamases that shield *S. pneumonia* and *H. sinfluenza* from ampicillin and amoxicillin respectively (Armbruster et al., 2010; Perez et al., 2014). Thus, to conclude, the biofilm structure and its architecture can change both the exposure of cells and the diffusion of drugs or antibiotics through them.

### 3.5 Mutations and altering cell walls via enzymatic process

Genomic mutations can lead to antibiotic resistance, even without strong selective stress or pressure. Mutations occur at a rate of 10^-10^-10^-9^ per nucleotide per generation in most bacteria (Woodford and Ellington, 2007; Schroeder et al., 2018). It has been reported that oxidative stress-causing agents can also accelerate the mutation rate, causing multidrug efflux pumps, mutagenesis, and resistance (Van Acker and Coenye, 2017). Defects in *mutS*, *mutL*, and *uvrD* genes can increase mutation frequency up to 100-fold according to the report (Leong et al., 1986; Schaaper and Dunn, 1987). Bacteria when possessing hypermutators, which can gain advantageous mutations under selection stress and may cause antimicrobial resistance, provided an excellent example of evolutionary mutations (Eliopoulos and Blazquez, 2003). This specific phenotype, which is resistant to ciprofloxacin and rifampicin, has also been reported in *Pseudomonas* biofilms (Driffield et al., 2008). In addition to the previously listed phenotypic traits, hypermutations have also been observed in *S. aureus* and *H. influenza* isolates from cystic fibrosis infections, but not in Enterobacteriaceae isolates from acute UTIs; this suggests that hypermutability is preferred in specific contexts (Prunier et al., 2003; Román et al., 2004; Kovacs et al., 2013). For bacteria like *E. faecalis* and *S. aureus* to produce biofilms, the *dltA* genes are essential (Gross et al., 2001; Fabretti et al., 2006). This has been shown by the reduction of vancomycin resistance in the strains of *S. aureus* following the deletion of the *dltA* gene. The dltABCD operon, crucial for D-alanylation of teichoic acid in gram-positive species was identified as a biofilm-specific gentamicin tolerance gene in streptococcus mutants, a dental pathogen causing infective endocarditis (Peschel et al., 2000; Neuhaus and Baddiley, 2003; Nilsson et al., 2016).

## 4 Exploring health conditions linked to the presence and impact of bacterial biofilm resistance

Roughly 80% of recurring and chronic microbial illnesses are caused by biofilms of bacteria. Biofilm-containing microbial cells have demonstrated 10–1000 times greater tolerance to drugs than planktonic cells. Infections linked to biofilms can be widely separated into two categories (Mah, 2012). Either biofilm might grow on the abiotic surfaces that include knee replacements, implants, dental units, catheters, contact lenses, screws, pins or prosthetic valves and joints or they are host tissue related that leads to chronic wounds, endocarditis, cystic fibrosis lings or chronic otitis media (Donlan, 2001; Burmølle et al., 2010). The infections related to the urinary tract and bloodstream are usually caused via a biofilm that initially developed on the medical implants associated with them and the only way to treat such infection is to get rid of those implants that not only raise the price of the therapy, but it also causes other health-related issues to the patients (Costerton et al., 2005; Sharma et al., 2019). Several of the primary infections associated with bacterial biofilms that are responsible for human illness are mentioned in **Table 1**.

**Table 1:** Microorganisms involved in biofilm-associated disease and their adherent surfaces.

Disease	Observation	Name of the microorganism	Surfaces	References
Chronic rhino sinusitis	Adherent biofilms on the sinus mucosa	*S. pneumoniae H. influenzae, S. aureus*,	Upper respiratory tract	Sanderson et al., 2006; Bezerra et al., 2011
Tuberculosis	Bacterial aggregates suspended in sputum; adherent bacteria in necrotic lung cavities (in mycobacterial infections)	*Mycobacterium tuberculosis*	Lungs	Zlosnik JE. et al., 2011
Cystic fibrosis	Adherent bacteria in necrotic lung cavities	*Burkholderia cepacia*	Lungs	Qvist et al., 2015
Urinary tract infection; kidney stones	Intracellular and extracellular clusters of bacteria in urine; matrix-encased clusters of bacteria within kidney stones and adhering to the stone surface	*P. aeruginosa*, *E. coli*, *P. mirabilis*, *Enterococcus* spp., *Staphylococcus* spp.	Urinary tract	Nickel et al., 1985; Rosen et al., 2007; Romanova et al., 2015
Chronic infection of cystic fibrosis patients; protracted bacterial bronchitis;	Bacterial aggregates suspended in sputum; adherent bacteria in necrotic lung cavities (in mycobacterial infections)	*P. aeruginosa*, *S. aureus*, *H. influenzae*, *M. catarrhalis*, *Mycobacteria* spp.	Lower respiratory tract	Bjarnsholt et al., 2009; DePas et al., 2016
Bacterial vaginosis	Bacterial aggregates in vaginal secretions; biofilms adhering to the vaginal epithelium	*Lactobacillus* spp., *G. vaginalis*, *F. vaginae*	Female reproductive tract	Swidsinski et al., 2005a; Hardy et al., 2015
Chronic prostatitis	Microcolonies adhering to the prostate ductal wall	*P. aeruginosa*, *E. coli*, *Staphylococcus* spp.	Male reproductive tract	Nickel and Costerton, 1993
Colorectal cancer, inflammatory bowel disease, post organ transplantation	Dense, adherent multispecies biofilms on the intestinal epithelium	*E. coli*, *R. gnavus*, *Bacteroides* spp., *F. nucleatum*	Colon	Swidsinski et al., 2005b; Baumgartner et al., 2021
Dental caries, periodontal disease	Highly structured multispecies biofilms collected from teeth	*Actinomyces* spp., *Streptococcus* spp., *Fusobacterium* spp., *Veillonella* spp.	Oral cavity	Zijnge et al., 2010; Welch et al., 2016
Chronic otitis media	Clusters of bacteria in aspirated secretions; adherent bacterial colonies on mucosal biopsies	*M. catarrhalis*, *H. influenzae*, *S. aureus S. pneumoniae*, *K. pneumoniae*	Middle ear	Hall-Stoodley et al., 2006; Homøe et al., 2009; Lee et al., 2009
Osteomyelitis	Thick, dense biofilms covering large areas of the bone surfaces	*S. aureus*, *P. aeruginosa*, *Streptococcus* spp.	Bone	Gristina et al., 1985; Johani et al., 2019
Peptic ulcers and gastric cancer	Extensive biofilms covering the gastric mucosa	*H. pylori*	Stomach	Carron et al., 2006; Coticchia et al., 2006
Delayed healing; predispositions include diabetes and severe burns	Bacterial aggregates embedded in the wound bed or on the wound surface	*S. aureus*, *P. aeruginosa*, *Enterobacter* spp., *Enterococcus* spp., *Proteus mirabilis*	Soft tissue wounds	James et al., 2008; Kennedy et al., 2010; Fazli et al., 2011
Infective endocarditis, atherosclerosis	Large clusters of bacteria encased within vegetations on heart valves; biofilm-like microcolonies within atherosclerotic arterial tissue and between the vascular smooth muscle and luminal plaque	*S. aureus*, *Enterococcus* spp., *Streptococcus* spp.	Cardiovascular system	Marrie et al., 1987; Mallmann et al., 2010; Lanter et al., 2014; Snow et al., 2016

## 5 Understanding the current impact of antibiotic resistance and its governing mechanisms

*S. aureus, S. pneumoniae, K. pneumoniae, P. aeruginosa, E. coli* and *E. faecium* are the most persistent and common multidrug-resistant bacteria that are linked to significantly high rates of death and morbidity worldwide (Dutt et al., 2022). Additionally, according to some reports, high levels of antibiotic resistance have also been linked to cancer-related neutropenia. Furthermore, because of biofilm biofilm-forming tendency of antibiotic-resistant bacteria, managing and treating newborn sepsis becomes a challenge in many clinical settings. In healthcare and hospital settings almost all procedures, surgeries, transplantation and intensive care are not typically carried out without antibiotics. However, with the increasing failure of first and second-generation antibiotics, the demands of expensive and time-consuming research for the next generation of antibiotics are increasing. Treating such resistant bacteria-mediated infections is complicated and requires the use of more costly and hazardous alternative treatments or higher doses (Magiorakos et al., 2012; Nesher and Rolston, 2014). Below we discuss some mechanisms that bacteria employ to confer antibiotic resistance:

### 5.1 Altering or safeguarding targets

Antibiotics are made with the specific intention of binding to their targets with a high degree of affinity and interfering with their regular activities. When targets modify structurally, antibiotics attach to them less effectively. Moreover, mutation also plays an important role in developing resistance by modifying the antibiotic target (e.g. the single nucleotide polymorphism mutation in the gene that codes the target). For instance, Rifampin resistance arises from an amino acid change in the *rpoB* gene. This mutation results in a decrease in rifampin binding affinity for its target, whereas transcription persists (Munita and Arias, 2016). In other bacteria like *streptococcus pneumonia*, the secretion of some binding proteins like penicillin-binding proteins (PBPs) lessens their target binding affinity with many beta-lactam antibiotics (Nagai et al., 2002). Moreover, antibiotic resistance can also be attained through post-translational alteration of genes that don’t include any mutation like the 16sRNA methylation in erythromycin ribosome methylase *(erm)* gene family leads to the modification of the drug binding sites that consequently prevents the binding of macrolides, lacosamide’s or streptogramin with 16sRNA (Kumar et al., 2014). Also, the A2503 residue methylation through chloramphenicol florfenicol resistance genes prevents and inhibits the binding efficiency of antibiotics like oxazolidinones, lacosamide, phenicol’s or stereograms with the target (23S rRNA) (Long et al., 2006).

### 5.2 Cell membrane or cell wall adaptation/alteration

In the case of Enterobacteriaceae, the bacterial membrane decreased permeability to carbapenems and that results in making them resistant to carbapenems antibiotics. Mosty in this family the resistance mechanism is mediated by the downregulation of porins (*OmpC* and *OmpF*) expression or due to its replacement by more selective porins or membrane channels (Baroud et al., 2013). Likewise, the lowered permeability of antibiotics like erythromycin, azithromycin, clarithromycin or azithromycin makes *V. cholerae*, *S. Enteric* and *P. aeruginosa*-like gram-negative bacteria resistant. Furthermore, by using efflux pumps bacteria also force antibiotics out into the extracellular matrix, which stops them from reaching their intended target (Wiese et al., 1999).

### 5.3 Ribosome protection

Certain bacteria develop a resistance mechanism called ribosome protection. As an inhibitor of bacterial protein synthesis, tetracycline causes the production of ribosome protection proteins by the bacteria, which attach to the ribosome target and stop tetracycline from binding to the ribosome (Roberts, 2005; Dutt et al., 2022). In these situations, the synthesis of ribosome protective proteins allows bacteria to proliferate despite the presence of tetracycline.

### 5.4 Enzymatic breakdown of antimicrobial substances

Antibiotics can also be rendered inactive by bacteria, which can likewise change their structural makeup and stop them from entering cells. This process is mainly through hydrolysis. Enzymes like chloramphenicol acetyltransferase or carbapenems degrade antibiotics including macrolides, aminoglycosides, phenicol as well as β-lactams (Dutt et al., 2022). Both the extended and early spectrum β-lactamases are active enzymes against β-lactams and oxyimino-cephalosporins (Lynch et al., 2013). TEM-1 β-lactamase and SHV-1 (sulfhydryl variable active site) enzymes, which are expressed by the plasmids in *E. coli* are good examples of such degrading enzymes that hydrolyse the multiple kinds of extended-spectrum cephalosporins. Furthermore, it is also reported that the modification or changes in the functional group of antimicrobials caused by degrading enzymes also contribute to antibiotic resistance.

## 6 Present treatment modalities targeting bacteria with biofilm-forming abilities

**Figure 2:**
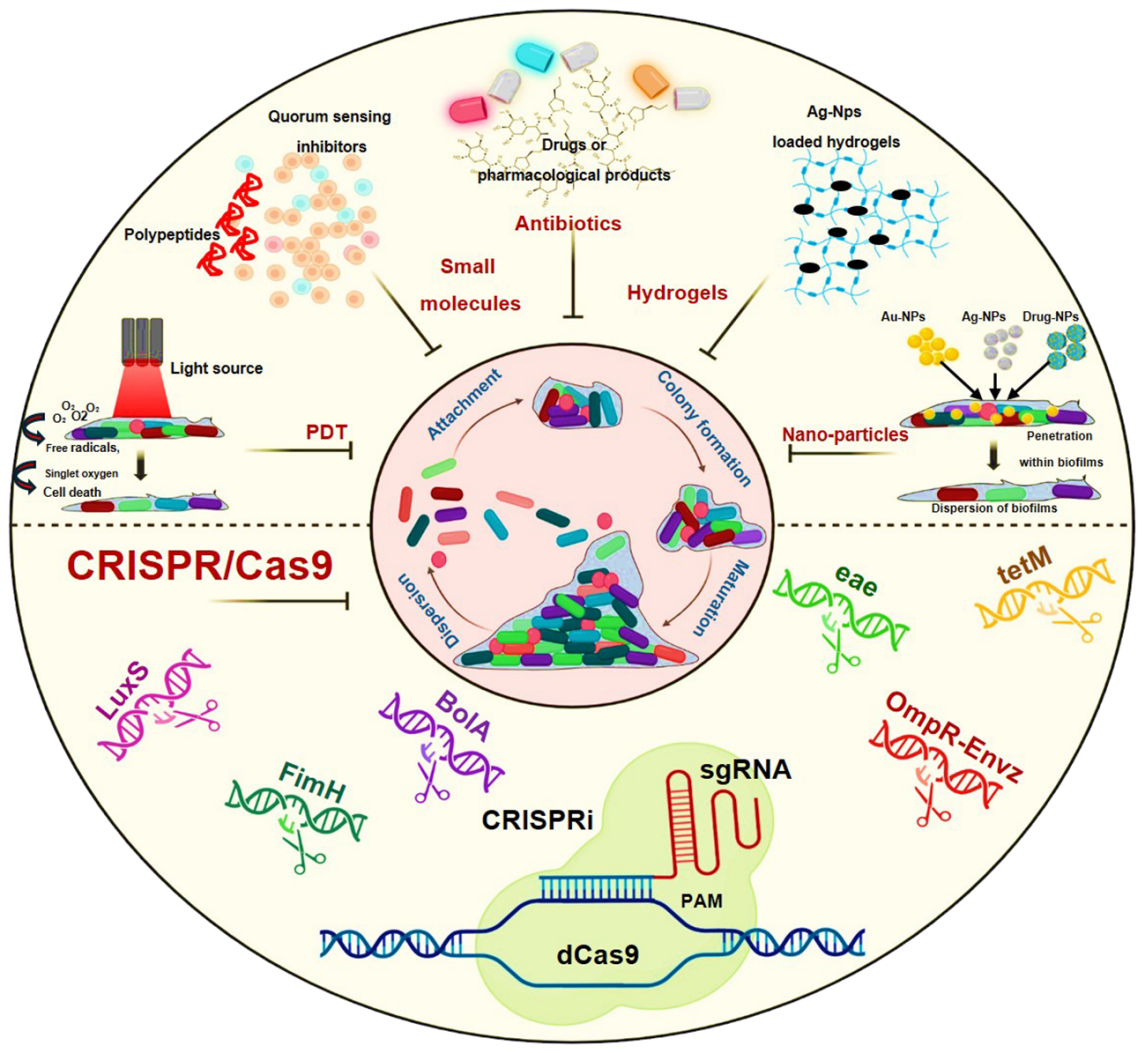
Schematic representation showing treatment modalities against biofilm-mediated infections: Photodynamic therapy (PDT), Small molecules as polypeptides or quorum sensing inhibitors, Antibiotics, Hydrogels, nanoparticles and the CRISPR/Cas9.

The fact that biofilm functions as a self-motivation mechanism during the pathogenic process helps to explain it. There have been several approaches and methodologies that have been employed to understand its antibiotic resistance nature and to target its residing bacteria. Some of its common approaches are the use of natural products, plant extracts, surface coatings, antibiotics, hydrogels, peptides, lasers during photodynamic therapy (PDT) and nanomedicines or nanoparticles (Hernández-Sierra et al., 2008; Lonn-Stensrud et al., 2008; Kolodkin-Gal et al., 2010; Hochbaum et al., 2011; Iannitelli et al., 2011; Kulshrestha et al., 2014; Muñoz-Egea et al., 2016; Misba and Khan, 2018; Misba et al., 2019). The dramatic portrayal of these treatment strategies i.e. Photodynamic therapy (PDT), small molecules as polypeptides or quorum sensing inhibitors, antibiotics, hydrogels and nanoparticles are shown in **Figure 2**.

The present treatment modalities utilize mainly the traditional methods to combat these biofilms that mainly focus on the dispersal of biofilm or its eradication or inhibition through antibiotics, small molecules inhibitors, enzymes, quorum sensing inhibitors, hydrogels etc (Dutt et al., 2022). **Table 2** shows the list of potential drug candidates and small molecules that are found effective in the mitigation of biofilm-related infections.

**Table 2:** Potential small compounds and drug candidates for biofilm inhibition.

Target	Compound or Drug Candidate	References
Inhibition of bacterial adhesins	3-(trimethoxysilyl)-propyldimethyloctadecyl ammonium chloride (QAS), vancomycin, zinc oxide and silver nanoparticles, iodine, copper, furanone, phloretin, oroidin	Ghosh, et al., 2020; Lee, et al., 2011; Kelly, et al., 2003
Nucleotide second messenger signaling systems/second messenger cyclic dimeric guanosine monophosphate/guanosine diphosphate (GDP) guanosine tetraphosphate (ppGpp), guanosine pentaphosphate (pppGpp), bis(30,50)-cyclic diguanylic acid (c-di-GMP), c-di-AMP, Rel enzyme, DGC	GSK- X9, terrain, saponin, vitamin C, sulfathiazole and azathioprine, LP 3134, LP 3145, LP 4010 and LP 1062, Amb2250085 and Amb379455, ebselen (Eb) and ebselen oxide (EbO), benzoisothiazolinone derivative, H19 and 925 (hiol-benzo-triazolo-quinazolinones), palmitic acid, and palmitoyl-dl-carnitine (pdlc)	Saiman, et al., 2003; Wenderska, et al., 2011; Sambanthamoorthy, et al., 2014; Zhou, et al., 2017
Diguanylate cyclase enzymes (DGCs)	Azathioprine, ebselen, sulfonohydrazide, sodium nitroprusside (SNP), S-nitroso-L-glutathione (GSNO), and S-nitroso-N-acetylpenicillamine (SNAP)	Ghosh, et al. 2020; Antoniani, et al., 2013; Barraud, et al., 2006
Unspecific	Bromoageliferin, TAGE (trans-bromoageliferin) and CAGE (cis-bromoageliferin), amburic acid, and 4-epi-pimaric	Ghosh, et al. 2020; Huigens, et al., 2007; Ali, et al., 2012
SpA, PIA, eDNA	5-methoxy2-[(4-methyl-benzyl)sulfanyl]-1H-benzimidazole (ABC-1)	Sambanthamoorthy, et al., 2011
Motility	Q24DA	Rasmussen, et al., 2011;
Biofilm formation/chaperone	Biphenylmannosides and dihydrothiazolo ring-fused 2-pyridone scaffold, bicyclic 2-pyridone, tetrazoles, acyl sulfonamides and hydroxamic acids (Mannocides/Pilicides), bicyclic b-lactams, dihydroimidazolo, and monocyclic 2-pyridone	Han, et al., 2010; Chorell, et al., 2010; Rasmussen, et al., 2011
Reduction in biofilm biomass and inhibition of enzyme activity	Cahuitamycin C,D,E, Auromomycin, Derivative 25, Skyllamycin A,B,C, Ebselen, Parthenolide, Ellagic acid, 3-β-xyl-EA, 3-α-ara-EA, Fiscetin, Hamamelitannin, Derivative 38, Amb 379455, LP3134, V-r8 and Congujate 7b	Trebino et al., 2021

Biofilm growth on implanted medical devices, prosthetic surfaces or biomaterials may be controlled by modifying the attachment surface, like coating the external surfaces. Many coating materials and biomaterials have been established, that make the target surface unfavourable for bacterial attachment. Moreover, the use of therapeutic agents and inhibitors against biofilm formation on dental implants or dental filling material has also demonstrated positive effects in combating biofilm-associated infections (Sharma et al., 2019).

As mentioned earlier too, the bacteria in biofilm form are more resistant and tolerant to antibiotics as compared to their planktonic form. Quorum sensing is a principal pathway that leads to biofilm-mediated bacterial maintenance and survival. Hence certain strategies that lead to the dispersal of biofilm or targeting quorum sensing mechanism have exhibited promising results in addressing biofilm-mediated infections (Lonn-Stensrud et al., 2008; McDougald et al., 2012; Guilhen et al., 2017; Roy et al., 2018; Sharma et al., 2019). Moreover, co-treatment or combination therapy that consists of antibiotics or drugs along with a biofilm dispersal agent has also demonstrated efficacy in these cases but has some limitations due to inappropriate concentration of both components and still research is going on (Barraud et al., 2006; Marvasi et al., 2014; Reffuveille et al., 2015; Roizman et al., 2017).

With the new research development and advancements in technologies, the future proposed methods to combat biofilm-mediated antibiotic infections include the use of nanoparticles, antimicrobial peptides, photodynamic therapy and implementation of gene editing technologies like CRISPR/Cas9 (**Figure 2**). Antimicrobial peptides are also considered as an alternative to antibiotics in eliminating biofilm-mediated infections, they are well-studied biofilm-eradicating agents (Flemming et al., 2008; Baltzer and Brown, 2011). They are ubiquitous in nature and cationic in nature. They consist of 5–90 amino acids (van Boxtel et al., 2017). Despite their exceptional potential to disrupt cell membranes, their mechanism of action is yet to be studied in the case of biofilms.

As reported, nanoparticles are also promising drug delivery systems that tend to penetrate deep due to their small size. Among all drug carriers, they are one of the most effective and explored drug carriers. In the case of biofilm-mediated infections, these particles enter the cells break the biofilm barrier and increase the availability of drugs or antibiotics to the bacterial cells. They have high efficacy, low toxicity, efficient penetration power and high site-specificity for drug release when they are given along with the drug (Hernández-Sierra et al., 2008; Harris et al., 2009; Kulshrestha et al., 2014).

In studies involving photodynamic therapy, some researchers have demonstrated its notable efficiency in fighting against biofilm-mediated antibiotic resistance, attributed to their tendency to generate reactive oxygen species (ROS) (Misba and Khan, 2018; Misba et al., 2019). On the other hand, considering gene editing technologies, numerous reports highlight the promising data regarding CRISPR/Cas9 and its derivatives like CRISPRi, in thwarting biofilms and their related infections (Zuberi et al., 2017a, Zuberi et al., 2017b; Azam et al., 2020; Zuberi et al., 2022). In the subsequent section, we will aim to delineate the potential avenues for the advancement of CRISPR/Cas9 and its barriers to overcome.

## 7 Anticipated opportunities, hypotheses and hindrances in utilizing CRISPR/Cas9 gene editing system to target bacterial biofilms

According to reports, CRISPR/Cas9 (Clustered regularly interspaced short palindromic repeat) exists approximately in 50% of bacterial genomes and 87% of archaeal genomes and has been acknowledged as an adaptive immune system in bacteria (Ran et al., 2015; Hille et al., 2018; Watson et al., 2021; Mayorga-Ramos et al., 2023). The CRISPR/Cas9 system has exhibited promising potential in recent years in the advancement and development of next-generation antimicrobial medicines or drugs to fight infections that are bought out by antibiotic resistance bacteria (Getahun et al., 2022; Mayorga-Ramos et al., 2023).

Targeting the genes that confer virulence and antibiotic resistance in bacteria has been a common application of this mechanism. CRISPR/Cas9 can be employed in two different ways: a pathogen-focused strategy and a gene-focused approach, depending on where the target gene is located (Li et al., 2016; Shabbir et al., 2018; Tang et al., 2019). Targeting chromosome regions to cause bacterial cell death is one pathogen-focused method. On the other hand, the gene-focused strategy includes focusing on the plasmids that may carry antibiotic-resistance genes (Palacios Araya et al., 2021; Nie et al., 2022). In such cases, the plasmid is eliminated, and the bacteria becomes antibiotic susceptible. The role of CRISPR/Cas9 has come across many times to target the genes that are implicated in antibiotic resistance (Nie et al., 2022; Tao et al., 2022). A study published by Bikard et al, used CRISPR/Cas9 to target the *mecA* gene (responsible for methicillin resistance) in USA300, the clinical isolates of *S. aureus*. His results revealed a marked decrease in the pollution of *S. aureus* in the mixed bacterial population as compared to the control group (Bikard et al., 2014).

In another research, a mouse skin colonization model was used to demonstrate that CRISPR/Cas9 was successful in specifically decreasing the colonization of Staphylococcus bacteria, in contrast to alternative treatment scenarios (Bikard et al., 2014; Wang et al., 2019). Moreover, in a separate study published by Ates et al., it was illustrated that resistance genes (*aacA, grlA, grlB* and *mecA*) in MRSA strains when targeted through CRISPR/Cas9 by designed CRISPR plasmids harbouring specific sgRNA, increase their susceptibility against antibiotics, hence changes their resistance profile (Juszczuk-Kubiak, 2024). Moreover, the use of pCasCure plasmids also came across in reversing the susceptibility of Enterobacteriaceae against carbapenems. pCasCure was reported successful in especially cleaving genes like *bal*_KPC_, *bla*_OXA-48_ and *bla*_NDM_ and targeting their corresponding plasmids (Hao et al., 2020).

The other team led by Yosef used the CRISPR/Cas9 system to eliminate plasmids containing *bla*_CTXM-15_ and *bla*_NDM-1_ (beta-lactamase genes) to eradicate *E. coli* that produce extended-spectrum beta-lactamases (ESBLs) (Yosef et al., 2015). The CRISPR/Cas system that targets are, the virulence factor in *E. coli* O157:H7 (EHEC), subsequently resulted in a 20-fold drop in viable cell counts, as shown by Citorik et al (Citorik et al., 2014). Rodrigues et al. tried to specifically eliminate the tetracycline (tetM)and erythromycin *(ermB)* in *E. faecalis* in both vitro and vivo conditions. His *in vivo* data demonstrated a considerable reduction in the percentage of antibiotic-resistant *E. faecalis* within the gut of mice (Rodrigues et al., 2019).

Askoura et al, revealed that *S. enterica* biofilm development, cell adhesion and cell invasion were impacted by CRISPR/Cas9 system that targeted sdiA (Askoura et al., 2021). Additionally, it’s noteworthy to mention the two of our studies that were published in 2017 manifesting the role of CRISPRi (CRISPR interference: derivative of CRISPR/Cas9) in restraining biofilm-mediated infections that are caused by clinical strains of *E. coli*. In one study we targeted *luxS*, the main quorum sensing gene in *E. coli* and in another article we tried to knockdown *fimH* gene and targeted bacterial adherence property through CRISPRi (Zuberi et al., 2017a, Zuberi et al., 2017b). Quorum sensing is one of the most important mechanisms that govern bacterial biofilm resistance against antibiotics, while the *fimH* gene plays a crucial role in bacterial virulence by contributing the fimbriae production. In both of our studies, CRISPRi demonstrated its highest level of effectiveness and exhibited optimal performance in targeting its specific genes and its related mechanisms, hence manifesting its lead role in biofilm-induced infections like urinary tract infections (UTI).

Additionally, in another study published by our group, we illustrated the role of CRISPRi in targeting the *bolA* gene (Azam et al., 2020). It has been discovered already that curli and fimbria formation have a role in the production of biofilms and are directly related to bacterial pathogenicity. *BolA* is a conserved protein and a transcriptional factor that is involved in bacterial motility and biofilm formation so by targeting this gene through CRISPR gene silencing we tried to combat these biofilm-mediated infections. We also targeted the *OmpR/EnvZ*, a two-component regulatory mechanism that is involved in transcriptional regulation when osmolarity changes. The main aim of that study was to elucidate the function of *OmpR/EnvZ* in controlling biofilm through curli and fimbriae production (Zuberi et al., 2022).

All our studies demonstrated exceptional outcomes and obtained outstanding success regarding the use of CRISPRi against biofilm-mediated infections. Not only this but these findings generated fresh insights and sparked innovative scientific discussion platforms regarding the use of this gene editing technology in combating biofilm-mediated infections, which represents a significant barrier within the realm of antibiotic resistance.

Analogous to the protective mechanism of thorns on a rose this gene editing technology also has certain intrinsic limitations that necessitate scientific scrutiny and consideration like the delivery challenge, which is the primary concern (Mayorga-Ramos et al., 2023). However, recent scientific researchers have suggested and hypothesized several different potential solutions to use this technology, such as employing the use of nanoparticles in conjugation with the CRISPR system (Li et al., 2018; Duan et al., 2021; Wan et al., 2021; Yan et al., 2021). Not only this but some studies have also proposed the concept of direct delivery of CRISPRi-edited bacterial cells to the infection site. This approach may facilitate the transfer of this gene editing mechanism to other virulent or antibiotic-resistant bacterial cells at the site of infection via natural conjugation of horizontal gene transfer mechanism (Ji et al., 2014; Watson et al., 2018; Barrangou et al., 2022).

## 8 Conclusion and future prospective

It is a known fact that Biofilm complicates the infections that are associated with communicable as well as non-communicable diseases. Other than that, the role of biofilm in post-operative infections, digestive disorders, cystic fibrosis, atherosclerotic arteries and infective endocarditis also has been documented in various studies and reports. Surface adherence is considered the prevailing mode of bacterial growth culminating in biofilm formation. Consequently, the biofilm environment fosters the emergence of antibiotic resistance or antimicrobial resistance. The understanding of how this lifestyle or environment influences the antimicrobial resistance evolution is still limited. Different hypotheses and explanations have emerged in this context like the proximity of bacterial cells in biofilms may facilitate the horizontal transfer and persistence of resistance genes within these bacterial populations.

The role of Biofilms in antibiotic resistance opens avenues and new vistas for repurposing existing drugs targeting biofilm. Forecasting antibiotic resistance by analysing the population of biofilm formers would be helpful in this scenario. Moreover, the use of nanoparticles in biomaterials and different drug delivery modes could also be considered as alternative options. Furthermore, to develop new and effective anti-biofilm agents computational and new sequencing technologies involving bioinformatic tools are also needed.

Currently, most of the studies focus on in silico screening, omics studies, and machine learning to identify the specific targets for anti-biofilm agents. To predicate antibiotic resistance and formulate therapeutic strategies it is essential to address potential protein targets, biofilm formation mechanisms and pathways. Integrated labs and computational science can lead to the development of successful anti-biofilm agents. RNA-Seq, a high-throughput technology, can assay gene regulation and expression, identifying transcriptomic signatures distinct to biofilms and bacterial dispersion.

As mentioned earlier, in addition to the advancements further research in the field of gene editing technology like CRISPR, is essential. It is imperative to address barriers in a CRISPR-like delivery system, as the advantages of utilizing this gene editing technology far outweigh its limitations, provided the delivery issue can be effectively resolved (Mayorga-Ramos et al., 2023). Moreover, through this mechanism, it could also become possible to target various genes associated with other pathways leading to bacterial biofilm formation and antibiotic resistance. For instance, genes responsible for efflux pumps could be the ones that can be specifically targeted, thereby enhancing our ability to combat antibiotic resistance.

## Author contributions

AZ: Software, Supervision, Writing – original draft, Writing – review & editing. NA: Conceptualization, Formal analysis, Investigation, Methodology, Supervision, Visualization, Writing – original draft, Writing – review & editing. HA: Validation, Writing – review & editing. MS: Methodology, Writing – review & editing. IA: Validation, Writing – review & editing.
